# Building bridges in the emergency room: educational alliances between junior residents and attending physicians

**DOI:** 10.3389/fmed.2025.1625760

**Published:** 2025-12-16

**Authors:** Satoru Yoshida, Chihiro Kawakami, Rintaro Imafuku, Osamu Nomura, Takuya Saiki

**Affiliations:** 1 Division of Medical Education, Graduate School of Medicine, Gifu University, Gifu, Japan; 2 Niigata City General Hospital, Niigata, Japan; 3 Medical Education Development Center, Gifu University, Gifu, Japan; 4 Nursing Research Promotion Center, Graduate School of Nursing, Nagoya City University, Nagoya, Japan

**Keywords:** educational alliance, junior residents, feedback, coaching, emergency room, qualitative research

## Abstract

**Introduction:**

Emergency rooms (ERs) offer valuable learning opportunities for junior residents, yet the time constraints and complexity of interprofessional collaboration often hinder the development of strong educational relationships with attending physicians. This study aimed to explore how junior residents perceive and build educational alliances with attending physicians during a short-term ER rotation, using the framework of the “educational alliance” (EA).

**Methods:**

We conducted semi-structured interviews with fourteen junior residents who completed a two-month ER rotation at an emergency center in Japan. Thematic analysis was applied using an inductive approach, guided by the three core components of the EA: shared goals, agreement on strategies, and development of mutual trust.

**Results:**

Nine themes emerged, which aligned with the three core EA components. Residents actively explored and shared their learning goals through both formal discussions and informal interactions with attendings, adjusting them as needed. While struggling with variations in clinical teaching strategies among attendings, residents developed their own styles through reflection and adaptation. Conversely, some residents experienced diminished autonomy due to unilateral instruction or felt conflicted between patient safety and educational challenges. Trust and appreciation toward attendings were formed progressively and were based on their attitudes, communication, and clinical judgment, contributing to psychological safety and deeper engagement.

**Conclusion:**

Despite time constraints, junior residents can gradually build educational alliances with attending physicians during short-term ER training. The quality of attending physicians’ engagement plays a critical role in supporting residents’ learning and relational development in fast-paced clinical settings.

## 1 Introduction

Emergency rooms (ERs) have long served as key educational settings where medical residents develop competencies in clinical decision-making, essential clinical procedural skills, and building relationships with patients and health professionals. However, ERs are busy and complex places with many patients whose diagnoses are uncertain, and thus teaching medical residents in the ER environment can be very challenging. Emergency care must be adaptive to provide optimal decision-making and treatment for patients from diverse backgrounds (1, 2). Attending physicians working in ERs must prioritize patient treatment over the instruction of medical residents when they are simultaneously managing multiple patients with critical conditions (3). Furthermore, interprofessional teams in the ER, including nurses, radiology technicians, laboratory technicians, and clinical engineers, require the attending physicians to take on a leadership role, further limiting the time available for direct teaching (4, 5). As a result, attending physicians are expected to assume a wide range of roles in emergency care, including serving as educators, clinical service managers, and team leaders, making it difficult to balance these competing responsibilities. This can make it difficult for them to maintain emotional well-being, make appropriate decisions, and dedicate sufficient attention to teaching responsibilities (6, 7).

Medical residents also often encounter significant challenges in ERs because they are newcomers to that environment and can have trouble establishing their role within the structured ER team. The trainee’s practice must be “supervised” and approved by the attending physicians; however, it is sometimes impossible for trainees to find an opportunity to consult with and seek feedback from the attending physicians. This may hinder the development of a mutual relationship between the trainee and the attending physicians (8).

It is imperative for attending physicians to provide effective feedback to medical residents. However, the time and opportunity for feedback dialogue between residents and attending physicians is often limited (1, 8), resulting in scarce interactions between residents and attending physicians in the ER. In surgery, for example, residents perceive that they do not receive as much effective feedback as their attendings believe they provide (9). Another study in emergency medicine suggested that residents are less satisfied with the feedback they receive than their attending physicians perceive (6). From a program perspective, it has been suggested that sharing learning goals through orientation sessions enables smooth transitions for residents (10, 11); however, this process is time-consuming, and there is a lack of consensus about its effectiveness (12).

Structured feedback and coaching from supervisors could produce certain educational outcomes (13). Telio et al. (14) have proposed the concept of the “educational alliance,” emphasizing that the relationship between supervisors and residents impacts the quality of feedback. The educational alliance is a belief about how learners perceive the educational relationship between learners and instructors. There are three core components of the educational alliance: first, the belief that there is mutual understanding of the purpose and goals of the relationship between the parties involved; second, the belief that there is agreement on how to work toward those goals and related activities; and third, the respect, trust, and appreciation toward the supervisors by the trainees, and the belief that these feelings are mutual (15). The ideal alliances that emerge from these partnerships can promote more effective educational retention and educational outcomes than the implementation of new teaching strategies. Some previous studies about the relationship between residents and attending physicians has demonstrated that residents lack confidence in setting goals for their training and seek support from their supervisors (16, 17), suggesting that residents frequently need to coordinate learning goals with their attending physicians. In addition, residents often set low learning goals, suggesting that mentorship for goal-setting may be beneficial for developing residents’ competencies (18). A qualitative study in psychiatry residency has found that the trust that residents feel toward their attending physicians influences their acceptance of feedback and subsequent co-regulated learning with attending physicians (15). Another mixed-methods study in critical care medicine illustrated that residents viewed having more prolonged contact with their attending physicians as increasing their autonomy and opportunities to obtain feedback; however, residents also felt that brief contact provided insufficient support and feedback from their attending physicians (19).

While the concept of the educational alliance -the educational relationship between attending physicians and residents—has gained increasing attention in clinical education, how residents perceive and experience the formation of this relationship in time-limited, high-pressure environments such as ERs remains underexplored in emergency medicine. This issue is particularly relevant in a system where many junior residents are still undecided about their future career paths and rotate through the ER for only a brief period. Prior studies have shown that providing meaningful feedback in such time-pressured environments is particularly challenging, with clinical workload and lack of time repeatedly identified as major barriers (20). Moreover, in emergency medicine, learners are required to move beyond fragmented advice and develop “feedback literacy” to productively engage with supervisory input (21). Episodic feedback also tends to reflect only the supervisor’s perspective, and a recent systematic review has highlighted that learners’ acceptance of feedback strongly depends on its relevance and credibility from their own perspective (22). These findings suggest that feedback is not merely a matter of information delivery, but rather a process shaped by goal alignment and the quality of the educational relationship between supervisors and residents. While other frameworks such as Team STEPPS (23), Edmondson’s work on psychological safety (24), and Scott’s (25) Radical Candor provide valuable insights into teamwork and communication, they do not directly conceptualize the supervisor–resident educational relationship. In contrast, the Educational Alliance (EA) framework explicitly addresses shared goals, negotiated strategies, and mutual trust, which are particularly critical in short-term ER rotations. For this reason, the Educational Alliance (EA) framework—which conceptualizes supervision as a collaborative process grounded in shared goals, negotiated strategies, and mutual trust—offers distinctive added value for examining feedback in the emergency setting. Yamamoto et al. indicated that residents perceived short-term emergency rotations as valuable for developing their professional identity and engaging in authentic participation (26). What remains insufficiently understood is how residents and supervisors actually negotiate goals, strategies, and trust within these constraints. Addressing this gap requires an explicit focus on the development of educational alliances in the ER. A better understanding of how junior residents develop mutual goals, agree on clinical strategies, and build trust with attending physicians will provide valuable insights for designing future educational interventions in emergency care settings. The purpose of this study is to explore how junior residents perceive the development of an educational alliance during their ER training. Specifically, the following research questions are addressed:

- How do junior residents describe and interpret their experiences of establishing mutual understanding of training goals and objectives in the ER?
- In what ways do junior residents perceive and negotiate training strategies with their attending physician during ER rotations?
- How do junior residents experience their feelings toward attending physicians, and how do these experiences shape the development of their relationships?

## 2 Methods

### 2.1 Qualitative research design

The study employed a qualitative research methodology, based on the constructivist paradigm, to conduct an in-depth analysis of residents’ complex perceptions of their attending physicians after 2 months of emergency medicine rotations. Fourteen face-to-face, semi-structured, in-depth, and open-ended interviews with junior residents were conducted and audio-recorded. Inductive thematic analysis was performed to identify the junior residents’ relational perceptions and feelings toward their attending physicians, with generative coding and theoretical interpretation by the research team.

### 2.2 Study subjects and setting

The participants in this study were postgraduate year one (PGY1) and postgraduate year two (PGY2) junior residents enrolled in Japan’s two-year clinical training program, which is mandatory for all medical graduates, regardless of their intended specialty. According to the system established by Japan’s Ministry of Health, Labour and Welfare, trainees must rotate through multiple specialties, including internal medicine, surgery, pediatrics, and emergency medicine (27). At our institution, a two-month emergency medicine rotation is included as part of this curriculum, and approximately 13–15 junior residents participate each year. During the emergency medicine rotation, junior residents engage in ER care on weekdays and are supervised by emergency physicians. During the study period, the ER held 14–15 attending physicians on staff. The attending physicians included board-certified emergency physicians and fellows. The supervising physicians were randomly assigned to ER clinic duty daily and worked with the junior residents.

After obtaining approval from the Research Ethics Committee, 14 participants (9 male and 5 female) agreed to participate in the study. The sample size was considered sufficient based on thematic saturation, defined pragmatically as the point when additional interviews no longer generated substantially new codes and the coding framework stabilized. We monitored this through coding logs and team discussions. We also drew on the concept of information power judging the sample adequate given our narrow aim (junior residents’ perceptions of educational alliances in ER), the homogeneity of participants, and the richness of the interview data (28). Details of the participants are shown in Table 1. The mean age of the participants was 26.1 years.

**Table 1:** Characteristics of the participants.

Participant code	Gender	Age	Specialty of interest
A	M	26	Gastroenterology
B	M	26	Cardiovascular internal medicine
C	F	25	Internal medicine
D	F	26	Obstetrics and gynecology
E	F	30	Emergency medicine
F	M	26	Internal medicine
G	M	29	Undecided
H	M	25	Gastroenterology
I	M	25	Internal medicine
J	M	27	Medical officer
K	M	25	Orthopedics
L	M	25	Gastroenterology
M	F	25	Cardiovascular internal medicine
N	F	25	Undecided

Participant codes, gender, age, and specialty of interest for the 14 junior residents interviewed in this study.

### 2.3 Data collection

Semi-structured interviews were conducted by the first author (SY) individually in a private room in a secure environment. Each interview lasted approximately 40–90 min, and the guide shown in Table 2 was used as a basis for the interview. Participants were asked about the following five key topics:

- The resident’s perceptions of shared goals of emergency department training with attending physicians;
- The resident’s perceptions of sharing emergency department training strategies and task performance strategies with attending physicians;
- Events that occurred between the resident and his/her attending physician that left an impression on the resident, as well as the attending physician’s attitude and what he/she said;
- The resident’s feelings toward the supervising physician and the supervising physician’s presumed feelings toward the resident; and
- The resident’s perceptions of building and transforming relationships with attending physicians.

**Table 2:** Interview guidance.

About training goals	
1. Tell me about your perceptions of sharing your training goals with your attending physicians.
2. Did the goals change over the course of the training?
3. If the goals changed, how did your attending physicians respond to these changes?
About training strategies	
4. Tell me about your perceptions of sharing your training strategies with your attending physicians.
About relationships with attending physicians	
5. How has your relationship with your attending physicians changed?
6. What else has happened with your attending physicians that has changed?
7. What are your feelings toward your attending physicians?
8. What feelings do you think your attending physicians have toward you?
9. If you feel trust in your attending physicians, in what ways do you trust them?
About feedback from attending physicians	
10. Have you ever received feedback that changed your own behavior or thinking?
11. When do you feel unwilling or unable to receive feedback from your attending physicians?

Key topics and sample questions used to explore junior residents’ perceptions of goals, strategies, relationships, and feedback during emergency room training.

This study was approved by the Ethics Committee of the Gifu University Graduate School of Medicine (2020–255) and was also approved by the Ethics Committee of the hospital (20-101).

### 2.4 Data processing

The interviews were audio-recorded, and a verbatim transcript was prepared. The first author anonymized personally identifiable information in the process. To ensure an audit trail in this study, all elements of the data analysis process (raw data, coded transcripts, researcher’s notes, and analysis results) were recorded in detail.

### 2.5 Data analysis

In this study, we used Braun and Clarke’s six-step thematic analysis in an inductive approach (29). In the first phase, all researchers systematically reviewed the verbatim transcripts and conducted a familiarization phase to deepen their understanding of the data. In the second phase, the textual data were broken down into smaller units, such as beliefs, actions, events, and thoughts, and SY and CK individually performed the initial coding. In the third phase, the initial codes were compared, and broader patterns of meaning (themes) were identified; SY and CK coded the remaining verbatim transcripts, describing the small units interpretively in terms of self-disclosure and integrating the data into more abstract themes. Although the thematic analysis was conducted inductively, Telio et al.’s (14) educational alliance model was used during the interpretive phase to help organize the themes under three conceptual elements: mutual understanding of goals, agreement on strategies, and trust in the supervisor. In this sense, the Educational Alliance framework served as a sensitizing concept rather than a predefined coding template (30). Thus, while our coding was inductive and grounded in the residents’ narratives, the interpretation was also informed by our theoretical orientation. Following Timmermans and Tavory (31) and Rinehart (32), this analytic stance is best described as an abductive approach, which reflects the iterative movement between empirical data and theoretical perspectives and enables the generation of theoretical insights grounded in participants’ experiences. In the fourth phase, all researchers iteratively reviewed the initial themes to ensure consistency with the data. In the fifth phase, each theme was analyzed in detail to establish a focus and determine the overall story of the theme. In the sixth phase, we discussed the relevance of the themes to the existing literature and reported the results of the study. The study was described in accordance with the Standards for Reporting Qualitative Research (SRQR) (33).

### 2.6 Trustworthiness

To improve the trustworthiness of the qualitative analysis, two researchers (SY and CK) independently coded and classified the data. Data interpretation and analysis were then cross-checked. The entire research team carefully reviewed the preliminary results multiple times to confirm the validity of the data analysis. In addition, member checks were performed on some participants to assess the validity of the researchers’ interpretations.

### 2.7 Theoretical framework: the educational alliance

The development of feedback research in medical education mirrors the progression of theoretical concepts in psychotherapy. In psychotherapy, the relationship between the two parties has been considered essential for the collaboration between therapist and patient aimed at achieving change. This dynamic is referred to as the “Theory of Therapeutic Alliance” (34). The hallmark elements of the therapeutic alliance encompass unity of goals, mutual consent to achieve these goals, and the bond between the therapist and the patient. The relationship between therapist and patient in psychotherapy bears a resemblance to the relationship between supervisors and trainees in clinical medical education, as both involve the provision of feedback to effect change in knowledge, self-concept, and behavior. Consequently, Telio et al.’s (14) study conceptualized the trainees as forming an educational alliance with the supervisors. Given the significance of the patient’s perception of the therapeutic alliance, it is essential to assess the educational alliance from the trainees’ perspective. In light of these considerations, Telio et al. (15) proceeded to define the elements constituting an educational alliance: the trainee’s belief that there is mutual understanding of the purpose and goals of the relationship; the learner’s belief that there is agreement on how to work toward those goals and related activities; the trainee’s trust and appreciation toward the preceptor, and the belief that these feelings are mutual; and the presence of a good relationship between the trainee and the preceptor, and that these feelings are reciprocal. In addition, trustworthiness as a clinician and as a clinical supervisor is essential. In Telio et al.’s (14, 15) study, it was further determined that perceptions of trustworthiness (i.e., the instructor’s motivation to teach), perceptions of the learner’s presence, and perceptions of feelings toward the learner (which tend to cluster around trust, respect, and goodwill) were significant factors (15). The study also placed emphasis on seeking evidence that learners feel a strong educational alliance with their supervisors. Within the framework of an educational alliance, trainees are expected to proactively explore and assess their supervisor’s commitment to the learning process from the initial encounter. This framework served as an interpretive lens for exploring how junior residents and attending physicians negotiated educational relationships during short-term emergency medicine training. It was particularly suited to this context because it conceptualizes teaching and learning as a reciprocal, relationship-centered process rather than a one-way transmission of knowledge. As Telio et al. (14) emphasized, feedback becomes meaningful and leads to effective learning only when grounded in a trusting educational alliance, which fosters learners’ receptivity and engagement. In the high-intensity and time-limited environment of emergency medicine, the ability to rapidly build mutual understanding, coordinate teaching strategies, and establish trust is essential for balancing patient safety and effective clinical training. Therefore, this framework provided a robust theoretical basis for interpreting how these relational elements evolve under the unique constraints of ER training.

## 3 Results

The analysis generated nine themes related to residents’ perceptions of the educational alliance (Table 3). Further detail on these themes is described below.

**Table 3:** Summary of the themes categorized by the three elements of the educational alliance.

Element 1	*Mutual understanding of the purpose and goal of the relationship*	
	Theme 1.1	Seeking learning goals and career goals as a junior resident
	Theme 1.2	Contingent goal-sharing
	Theme 1.3	Change in goals through feedback from the attending physician
Element 2	*Agreement about how to work toward relationship goals*	
	Theme 2.1	Recognizing and being puzzled by the varying strategies of different attending physicians
	Theme 2.2	Resentment of teaching strategies that do not respect junior resident autonomy
	Theme 2.3	Conflict between educational challenges and the strategy of prioritizing medical safety
Element 3	*The learner’s respect, trust, and appreciation toward the attending physician*	
	Theme 3.1	Favors and trust won from the attending physician
	Theme 3.2	Trust and respect toward the attending physician formed while struggling between dependence and independence
	Theme 3.3	Bond between the resident and the attending physician

Summary of the nine themes identified in the analysis, organized under the three core elements of the Educational Alliance framework: (1) mutual understanding of goals, (2) agreement on strategies, and (3) respect, trust, and appreciation toward attending physicians.

### 3.1 Mutual understanding of the purpose and goal of the relationship

#### 3.1.1 Seeking learning goals and career goals as a junior resident

When asked by their advisors about the goals of emergency training, and the intertwining of “what I have to do as a physician” with “my own future goals,” the junior residents became keenly aware of the need to explore not only their learning goals as junior residents but also their career goals as physicians. In addition, the junior residents noted the variation among attending physicians in terms of how well attendings understood and shared residents’ emergency training goals based on their intended future specialties, previous experiences, and desire for independent practice of residents. However, the junior residents perceived a disconnect between their own learning needs and the content of the instruction they received, and perceived insufficient support for their development. On the other hand, when they felt that their goals were shared through conversations and reflection aimed at goal alignment with attending physicians, the junior residents were motivated to further enhance their goals. Even when goals were not fully shared, the junior residents were grateful for the guidance they received from their attending physicians.

*I am often asked by the attending doctor what goal I want to achieve during this training. I talked to him quite a bit at that point, and he understood (the goal I want to achieve). I felt that (the attending doctor) was giving me guidance according to my goal.* (Resident C)

*Personally, I felt that there was a slight discrepancy between my initial perception and the direction I had to take and what I was doing.* (Resident M)

#### 3.1.2 Contingent goal-sharing

The junior residents felt that their ability to share their own goals for the duration of their residency depended on their attending physician’s response. In other words, while they appreciated the structure that allowed them to share their goals with their attending physicians during formal orientation, they felt that the opportunities for their attending physicians to question them about their supposedly shared goals were often informal situations. They recognized that such opportunities sometimes arose from casual conversation, and sometimes they were rarely questioned. As a result, the junior residents perceived that building relationships sometimes proceeded with ambiguous shared goals. While the junior residents lamented their attending physicians’ lack of interest in the junior residents’ goals, in retrospect, they were acutely aware that they themselves needed to be proactive in communicating their goals. *Every time I was on duty, I was paired with different or* var*ious attending doctors, so I was not able to share (my goals) with all of them.* (Resident I).

*I honestly thought that (the objectives of the training) were a bit haphazard.* (Resident J)

#### 3.1.3 Change in goals through feedback from the attending physician

The junior residents perceived that their goals became more specific as they gained field experience. When they received feedback from attending physicians that facilitated goal adjustment, they perceived greater progress toward achieving their goals. However, they also recognized that it was difficult for attending physicians, working in a busy ER setting with numerous residents at varying levels, to tailor goals to each resident’s growth.

*That (my goals) will change… so I probably haven’t done much of that (adjusting goals to suit myself). It is difficult for residents to achieve that (adjusting goals to suit oneself). I think some teachers can do it (tailor-made adjustment of goals) and some cannot*. (Resident N)

### 3.2 Agreement about how to work toward relationship goals

#### 3.2.1 Recognizing and being puzzled by the varying strategies of different attending physicians

The junior residents were greatly perplexed when they were assigned tasks by their attending physicians, as the content and difficulty level of the tasks varied from one attending physician to another. When their attending physicians did not explicitly share their clinical strategies, they recognized that they had to guess their attending physicians’ intentions and expected behavior. Therefore, they recalled that they often felt stressed. In addition, because each attending physician had a unique style of medical treatment strategy, the junior residents sought the best strategy while listening to their attending physicians’ opinions to avoid being misjudged or appearing to have made a mistake. Upon reflection, several junior residents noted that exposure to different attending physicians’ strategies broadened their own approaches and helped them begin to form their own practice style.

*How I should behave (in the ER) depends on the attending physicians, and how much they leave things to the residents. I have never specifically asked to what extent (the attending physician) would leave it to the resident.* (Resident H)

*If I don’t know if there are mines, I’m a bit scared to walk, but if I know that there are mines here and that it’s safe here, I can walk because I can learn from walking.* (Resident F)

*I can hear (feedback) from* var*ious attending physicians, so I can learn that there are other ways of thinking about similar cases.* (Resident J)

#### 3.2.2 Resentment of teaching strategies that do not respect junior resident autonomy

The junior residents felt that they sought to learn independently in the clinical setting, and they recognized that they could only grow through examining patients on their own. They also felt that sharing clinical strategies, such as instructions on how to proceed and the specifics of tasks and estimated time required, helped them to feel more comfortable and engaged in the practice. However, when the attending physician unilaterally took control of the flow of medical treatment and proceeded without asking the junior resident’s opinion, or when he or she repeatedly confirmed the junior resident’s findings and omitted the diagnostic process, the junior residents felt helpless, and their self-esteem was damaged. The junior residents also perceived that being deprived of independent experience would turn learning into mere business work.

*I was a bit scared at the beginning because I didn’t know what to do, I couldn’t make any initial moves.* (Resident I)

*I got more opportunities where the attending physician would let me take the lead like, “I’ll give you 15 minutes—go ahead and do the exam and report back to me.” Personally, when I’m given that chance to handle it on my own, it makes me really happy. I feel like they are respecting my autonomy—and that means a lot to me.* (Resident I)

*The attending physicians don’t listen to me. They didn’t even ask me how I was.* (Resident E)

*Some of the attending physicians don’t even wait for us (to see the patient)… and even if we were seeing the patients together, I was just touching the patient, and… the supervising doctors would already have the same findings (and I would have the same findings as the supervising doctor). The supervising physician would just tell the residents (‘Well, let’s order this examination’) Well… (I said to myself) I don’t need to be there….* (Resident D)

#### 3.2.3 Conflict between educational challenges and the strategy of prioritizing medical safety

Initially, the junior residents recognized that, as learners, they would ideally like to grow by being assigned a task to test their abilities in an environment where failure is tolerated. However, through the experience of trial and error in training, including the educational delegation of various tasks by various attending physicians, the junior residents became aware that patient safety was the top priority as a medical professional. The junior residents also understood that the attending physicians also face the conflict between careful practice and the need to provide educational training. The junior residents recognized that through the experience of exploring their own practice strategies with their attending physicians, they would learn to behave in a mature and respectful manner as junior residents.

*Of course, I would be more grateful if I had more opportunities to test my abilities on my own, but I think there are probably cases where it is to the detriment of the patient for a resident to try things out on his/her own… (There was an error that I missed before.) I reflected on it a lot, but I think it leads to that kind of growth, and… growth and danger (medical safety) are two sides of the same coin… I think it’s true that attending physicians don’t have the luxury of running the ER in a very safe way, while also encouraging the growth of the residents. I think that is inevitable.* (Resident N)

### 3.3 The learner’s respect, trust, and appreciation toward the attending physician

#### 3.3.1 Favors and trust won from the attending physician

The junior residents, especially at the beginning of their training, were painfully aware of the low expectations and trust that their attending physicians had for them, which they recognized from their attending physicians’ attitudes and speech. If the junior resident was seen only as an object of evaluation and felt that the attending physician did not trust him or her, the junior resident’s favorable impression of the attending physician was greatly reduced, and he or she sometimes felt disappointed. On the other hand, even when the ER was busy and the atmosphere was tense, the junior residents felt reassured by the fact that their attending physicians treated them fairly and with respect. They also sometimes felt that they were more favorably accepted by their attending physicians when their desired specialty matched their attending physician’s specialty. The junior residents perceived that gaining their attending physicians’ trust was associated with their own growth and motivation. As their relationships with attending physicians developed, they tended to participate more actively in clinical practice.

*(I) felt that (the attending physician) must have thought that I was useless… (The attending physician) doesn’t say it, but his eyes say it. (I feel it). I feel I am being criticized (for my own existence) … To be liked… you have to be competent.* (Resident D)

*I don’t feel (any) pressure at all (from the attending physician) if (the attending physician) is not overbearing. Because the attending physicians are (emotionally) constant.* (Resident D)

*I’ve had quite a few times when the attending physician gives me (feedback), if I’m considering emergency medicine (as my career), even though he probably doesn’t give it to other people maybe he likes me and is giving me this feedback.* (Resident L)

*After the relationship with the supervisor has been established, I think that rather than receiving feedback, I have been studying more on my own because I want to grow in these areas.* (Resident J)

#### 3.3.2 Trust and respect toward the attending physician formed while struggling between dependence and independence

The junior residents wanted to be able to practice independently as clinicians. On the other hand, they also wanted to be observed by their attending physicians as learners. The junior residents were gradually growing while struggling between their dependence on and currying favor with their attending physicians and their pride in their independence. The junior residents described that witnessing their attending physicians manage critically ill patients, follow up on their errors, and teach with passion helped them develop trust and respect. The junior residents perceived that their increased clinical and educational trust in their attending physicians accelerated their liking for them. This led to psychological safety and deep engagement to training, which further deepened their trust in and liking for their attending physicians.

*I felt that I could trust my advisor when I saw how he handled situations where the patient’s vital signs were unstable and he was in a hurry, but he was able to deal with the situation accurately. I felt that (the attending physician) was trustworthy because I felt safe working with him.* (Resident A)

*I’ve come to know that there is a sense of security that if I have a problem, the attending physician will help me. In the end, I feel that (the attending physician) will do something about it.* (Resident M)

#### 3.3.3 Bond between the junior resident and the attending physician

The junior residents were torn between tension and intimacy during the training period, trying to find the appropriate distance between the attending physician and themselves. They hoped to reduce stress as much as possible and build a better relationship. In the process, the junior residents began to realize that a bond was gradually developing between them, even though they were initially just performing their duties.

*I think that a relationship where you only talk about clinical issues is not very close, and I’m very happy to be able to talk about private matters with the attending physicians. I think that a relationship where you can talk about such (social) things has to be much closer.* (Resident B).

## 4 Discussion

**Figure 1:**
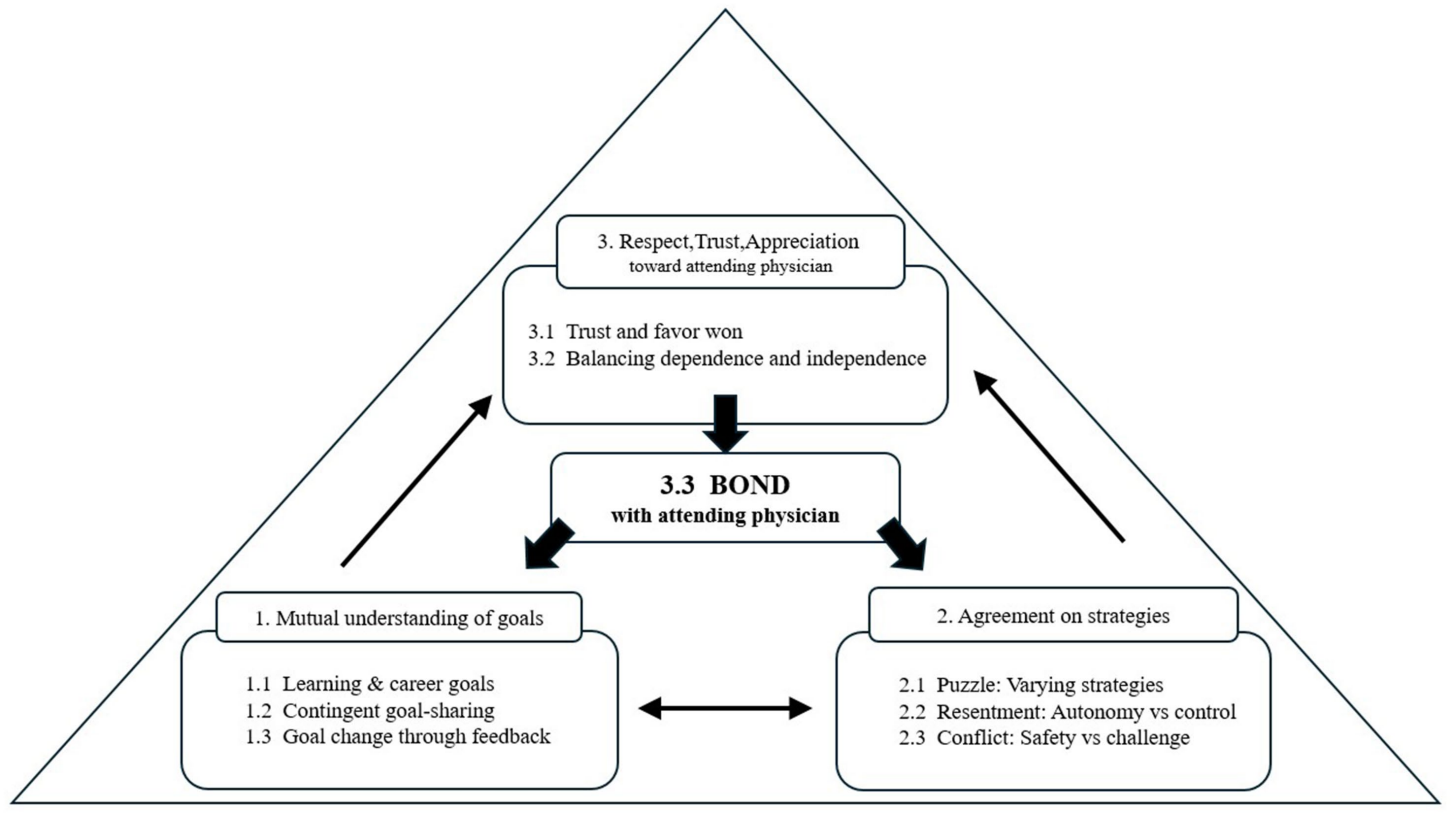
“Process model of educational alliance formation: thematic description.” The model integrates nine themes into three core elements (1: Mutual understanding of goals; 2: Agreement on strategies; 3: Respect, trust, and appreciation toward attending physicians), illustrating how goals, strategies, and trust interact dynamically in a cyclical process that accelerates alliance formation.

This study explored how junior residents perceive the development of the educational alliance during emergency department training. Our findings suggest that the formation of this alliance is a dynamic and cyclical process in which residents and attending physicians progressively establish shared goals, negotiate strategies, and cultivate mutual trust and appreciation. Through these reciprocal interactions, residents come to balance dependence with autonomy and to strengthen their relational bond with attending physicians, fostering a learning environment characterized by both psychological safety and professional growth (Figure 1). Our model is grounded in Telio et al.’s (14, 15) conceptualization of the Educational Alliance, which adapts Bordin’s (34) working alliance model in psychotherapy. Building on this foundation, our findings extend the relational perspective into the emergency training context. Unlike prior research on feedback or supervision that examined these elements in isolation, our model highlights their cyclical interdependence: trust and bonding emerge from goal-setting and strategy coordination, while simultaneously reinforcing them, thereby creating a self-perpetuating loop that accelerates alliance formation. This perspective provides added value by framing feedback and supervision not merely as discrete educational practices, but as relational processes that consolidate an alliance under the constraints of short-term ER training.

Telio et al. (14, 15) shed light on the importance of alliance-building, shared goals and strategies, and mutual trust from the perspective of the educational alliance. In the context of educational alliances, this study illustrates how the act of exchanging ideas about training approaches as part of their clinical practice influences the alliance between attending physicians and junior residents. Prior research has suggested that patient characteristics, medical safety, and junior resident competence influence the formation of an effective ER training program (6, 8, 19, 35, 36). This study specifically found that interactive discussions about goals-setting and strategies, which helped develop their feelings toward the attending physician, had a continuous impact on junior resident performance.

### 4.1 Conversations about junior residents’ goal-setting and coordination

As Themes 1.1 through 2.3 demonstrated, clarifying goals and coordinating strategies were central to initiating and sustaining alliances. Nothnagle et al. (16) noted that junior residents lack confidence in their abilities in self-regulated learning skills such as goal setting. Sawatsky et al. (18) further showed that the quality of learning goals does not automatically improve with clinical experience. Our findings extend these perspectives by showing that informal conversations—beyond structured orientations—provided critical opportunities for alignment. These micro-interactions created space for residents to feel heard and for supervisors to tailor support. This suggests that alliance-building requires attention not only to formal curriculum design but also to everyday dialogues in clinical practice.

### 4.2 Progressive independence

As Themes 2.3 and 3.2 demonstrated, residents experienced ambivalence between autonomy and patient safety. Progressive independence refers to the gradual process by which junior residents become capable of providing patient care independently, with the level of supervision decreasing as their competence grows. While supervision is traditionally framed as a safeguard for safety, concerns have been raised about the potential impact of compromising progressive independence (37). Our findings highlight the reciprocal nature of progressive independence and alliance-building: residents interpreted supervisory adjustments as signals of trust, not merely restrictions. Trainees may become frustrated when attending physicians emphasize only medical safety without recognizing the importance of progressive independence. Moreover, confusion about assigned tasks and roles has been shown to be a source of stress in the workplace (38) and a potential contributor to burnout (39). This suggests that supervisors should consciously calibrate independence to simultaneously advance learning and strengthen the alliance—particularly in the ER, where rapid decision-making and varying supervisory styles can otherwise exacerbate frustration and fatigue.

### 4.3 Assessment and alliance

As Themes 3.1 (and partly 3.2) demonstrated, evaluation functioned as both a barrier and a motivator for alliance-building. In line with Dweck’s Theory of goal orientation on learning (40, 41), evaluation can shape how residents engage with attending physicians. Huffman et al. reported that internal medicine residents who are preoccupied with favorable evaluation tend to avoid discussions with their supervisors, thus missing valuable growth opportunities (42). Our findings add nuance: once trust was established, residents shifted their focus from “being evaluated” to personal growth, actively approaching supervisors for guidance. This shift suggests that educational alliances can transform *evaluation* from a source of anxiety into a platform for deeper engagement and learning, thereby fostering residents’ willingness to seek feedback and deepen the alliance.

### 4.4 Psychological safety and alliance

As Theme 3.3 demonstrated, psychological safety played a central role in alliance formation. Our findings indicate that when residents felt psychologically safe, they were more willing to seek feedback from their attending physicians. At the same time, a certain degree of tension can sustain motivation to take on challenges and prevent complacency. This balance suggests that educational alliances foster both safety and constructive challenge, enabling residents to engage fully in the ER learning environment. These results are consistent with prior work: a mixed-method study of team dynamics showed that when psychological safety was absent, members with less hierarchical power did not feel free to speak up, highlighting the importance of intimacy and familiarity between leaders and team members (43). Similarly, a study of surgical training found that increased psychological safety enhanced residents’ awareness of supervisory support and willingness to take advantage of learning opportunities (44). At the same time, Eldor et al. demonstrated that excessive psychological safety may reduce performance (45). Our findings align with these perspectives by showing that although residents valued goodwill and closeness with their attendings, they did not seek intimacy as an end in itself; rather, they valued such relationships insofar as they fostered educational dialogue and promoted growth.

### 4.5 Implications for practice and policy

Our findings have several implications for postgraduate training in emergency medicine. At the program level, structured opportunities for goal-sharing early in the rotation may help align expectations despite frequent supervisor changes. For faculty development, attendings may benefit from training to explicitly communicate their clinical strategies and to recognize that supervisory adjustments are interpreted by residents as signals of trust. At the policy level, supervision structures should balance patient safety with progressive independence, ensuring that residents can gradually assume responsibility while maintaining psychological safety. These implications extend beyond existing feedback frameworks by emphasizing that feedback is embedded in a broader relational process of alliance-building, in which goals, strategies, and trust interact dynamically.

### 4.6 Limitations

It should be noted that this study has several limitations. First, the data collection was based on the theoretical framework of the educational alliance for designing the interview guide and analysis of the data, and this theoretical position may have had some influence on the generation of themes. In order to increase the reliability of the analysis, several researchers were involved in the analysis process and iteratively examined the validity of the elaboration and interpretation of the themes. Second, characteristics of this setting—short duration, multiple supervisors, and non-continuous supervisory relationships—are not unique to the ER. Similar dynamics can also be observed in outpatient clinics, brief subspecialty rotations, and on-call shifts, suggesting a degree of transferability of our findings beyond the ER. At the same time, educational alliances are inevitably shaped by institutional and cultural contexts, and additional studies in diverse environments will be necessary to establish the robustness and applicability of these insights. Third, the study focused on the narratives of trainees and did not include the perspectives of attending physicians or other health professionals in the ER medical team, which may give a one-sided view of the whole relationship. However, to our knowledge, no previous qualitative studies have examined ER training from the residents’ perspective using the EA framework. Therefore, despite this limitation, our findings provide a valuable contribution by illuminating how residents themselves experience and construct educational alliances in this unique context. Fourth, the participants’ narratives were based on their retrospective interpretation of their own experiences. As such, the findings may not fully reflect their perceptions during the actual training, and may have been influenced by reconstructed memories or social desirability bias.

## 5 Conclusion

This qualitative study highlights how junior residents’ perceptions of their educational alliance with their attending physicians impact their learning during emergency training. The findings on junior resident perceptions in this study provide insights into the process and influence of the educational alliance for attending physicians. This study will help attending physicians to understand how they can develop mutual goals, agree on clinical strategies, and build trust with junior residents when designing future educational interventions in emergency care settings.

## Data Availability

The raw data supporting the conclusions of this article will be made available by the authors, without undue reservation.

## References

[1] Aldeen AZ Gisondi MA. Bedside teaching in the emergency department. Acad Emerg Med. (2006) 13:860–6. doi: 10.1197/j.aem.2006.03.557, 16766739

[2] Derlet RW Richards JR. Overcrowding in the nation’s emergency departments: complex causes and disturbing effects. Ann Emerg Med. (2000) 35:63–8. doi: 10.1016/S0196-0644(00)70105-3, 10613941

[3] Atzema C Bandiera G Schull MJ. Emergency department crowding: the effect on resident education. Ann Emerg Med. (2005) 45:276–81. doi: 10.1016/j.annemergmed.2004.12.011, 15726050

[4] Wilson KM Leeman J Saunders B Havens DS. Improving physician engagement in interprofessional collaborative practice in rural emergency departments. J Interprof Educ Pract. (2018) 11:51–7. doi: 10.1016/j.xjep.2017.12.005

[5] Paquin H Bank I Young M Nguyen LHP Fisher R Nugus P. Leadership in crisis situations: merging the interdisciplinary silos. Leadersh Health Serv (Bradf Engl). (2018) 31:110–28. doi: 10.1108/LHS-02-2017-0010, 29412098

[6] Yarris LM Linden JA Gene Hern H Lefebvre C Nestler DM Fu R . Attending and resident satisfaction with feedback in the emergency department: feedback in the ed. Acad Emerg Med. (2009) 16:S76–81. doi: 10.1111/j.1553-2712.2009.00592.x 20053217

[7] Shayne P Lin M Ufberg JW Ankel F Barringer K Morgan-Edwards S . The effect of emergency department crowding on education: blessing or curse? Acad Emerg Med. (2009) 16:76–82. doi: 10.1111/j.1553-2712.2008.00261.x, 18945243

[8] Chaou C-H Monrouxe LV Chang L-C Yu S-R Ng C-J Lee C-H . Challenges of feedback provision in the workplace: a qualitative study of emergency medicine residents and teachers. Med Teach. (2017) 39:1145–53. doi: 10.1080/0142159X.2017.1366016, 28830288

[9] Jensen AR Wright AS Kim S Horvath KD Calhoun KE. Educational feedback in the operating room: a gap between resident and faculty perceptions. Am J Surg. (2012) 204:248–55. doi: 10.1016/j.amjsurg.2011.08.019, 22537472

[10] Hiraoka M Kamikawa G McCartin R Kaneshiro B. A pilot structured resident orientation curriculum improves the confidence of incoming first-year obstetrics and gynecology residents. Hawaii J Med Public Health. (2013) 72:387–90. 24251084 PMC3831566

[11] Mank VMF Wiggins A Lowe D Breighner C. Evaluation of an education-based training orientation for resident physicians in an intensive care unit in Hawai’i. Hawaii J Health Soc Welf. (2022) 81:223–7. doi: 10.1542/peds.2016-1823, 35923384 PMC9344538

[12] McGrath J Barrie M Way DP. Emergency medicine resident orientation: how training programs get their residents started. West J Emerg Med. (2017) 18:97–104. doi: 10.5811/westjem.2016.10.31275, 28116017 PMC5226773

[13] Sargeant J Lockyer J Mann K Holmboe E Silver I Armson H . Facilitated reflective performance feedback: developing an evidence- and theory-based model that builds relationship, explores reactions and content, and coaches for performance change (R2C2). Acad Med. (2015) 90:1698–706. doi: 10.1097/ACM.0000000000000809, 26200584

[14] Telio S Ajjawi R Regehr G. The “educational alliance” as a framework for reconceptualizing feedback in medical education. Acad Med. (2015) 90:609–14. doi: 10.1097/ACM.0000000000000560 25406607

[15] Telio S Regehr G Ajjawi R. Feedback and the educational alliance: examining credibility judgements and their consequences. Med Educ. (2016) 50:933–42. doi: 10.1111/medu.13063, 27562893

[16] Nothnagle M Anandarajah G Goldman RE Reis S. Struggling to be self-directed: residents’ paradoxical beliefs about learning. Acad Med. (2011) 86:1539–44. doi: 10.1097/ACM.0b013e3182359476, 22030764

[17] Lockspeiser TM Li S-TT Burke AE Rosenberg AA Dunbar AE 3rd Gifford KA . In pursuit of meaningful use of learning goals in residency: a qualitative study of pediatric residents. Acad Med. (2016) 91:839–46. doi: 10.1097/ACM.0000000000001015, 26630605

[18] Sawatsky AP Halvorsen AJ Daniels PR Bonnes SL Issa M Ratelle JT . Characteristics and quality of rotation-specific resident learning goals: a prospective study. Med Educ Online. (2020) 25:1714198. doi: 10.1080/10872981.2020.1714198, 31941433 PMC7006652

[19] Miller KA Cavallaro SC Dorney K Hirsch A Monuteaux M Nagler J. Paths to learning: how residents navigate transience in supervisory relationships in the emergency department. AEM Educ Train. (2024) 8:e11037. doi: 10.1002/aet2.11037, 39493701 PMC11527735

[20] Reddy ST Zegarek MH Fromme HB Ryan MS Schumann S-A Harris IB. Barriers and facilitators to effective feedback: a qualitative analysis of data from multispecialty resident focus groups. J Grad Med Educ. (2015) 7:214–9. doi: 10.4300/JGME-D-14-00461.1, 26221437 PMC4512792

[21] Noble C Young J Brazil V Krogh K Molloy E. Developing residents’ feedback literacy in emergency medicine: lessons from design-based research. AEM Educ Train. (2023) 7:e10897. doi: 10.1002/aet2.10897, 37529173 PMC10387830

[22] Dai CM Bertram K Chahine S. Feedback credibility in healthcare education: a systematic review and synthesis. Med Sci Educ. (2021) 31:923–33. doi: 10.1007/s40670-020-01167-w, 34457934 PMC8368112

[23] TeamSTEPPS®. Strategies and tools to enhance performance and patient safety Agency for Healthcare Research and Quality; Department of Defense, Rockville (2006).

[24] Edmondson A. Psychological safety and learning behavior in work teams. Adm Sci Q. (1999) 44:350–83. doi: 10.2307/2666999

[25] Scott K. Radical candor: be a kick-ass boss without losing your humanity. New York, NY: St. Martin’s Press (2017).

[26] Yamamoto I Obara H Verstegen D. How do mandatory emergency medicine rotations contribute to the junior residents’ professional identity formation: a qualitative study. BMC Med Educ. (2024) 24:1054. doi: 10.1186/s12909-024-06051-4, 39334029 PMC11429119

[27] Nishigori H. Medical education in Japan. Med Teach. (2024) 46:S4–S10. doi: 10.1080/0142159X.2024.2372108, 39545499

[28] Malterud K Siersma VD Guassora AD. Sample size in qualitative interview studies: guided by information power: guided by information power. Qual Health Res. (2016) 26:1753–60. doi: 10.1177/1049732315617444 26613970

[29] Braun V Clarke V. Reflecting on reflexive thematic analysis. Qual Res Sport Exerc Health. (2019) 11:589–97. doi: 10.1080/2159676X.2019.1628806

[30] Blumer H. What is wrong with social theory? Am Sociol Rev. (1954) 19:3. doi: 10.2307/2088165

[31] Timmermans S Tavory I. Theory construction in qualitative research: from grounded theory to abductive analysis. Sociol Theory. (2012) 30:167–86. doi: 10.1177/0735275112457914

[32] Earl Rinehart K. Abductive analysis in qualitative inquiry. Qual Inq. (2021) 27:303–11. doi: 10.1177/1077800420935912

[33] O’Brien BC Harris IB Beckman TJ Reed DA Cook DA. Standards for reporting qualitative research: a synthesis of recommendations. Acad Med. (2014) 89:1245–51. doi: 10.1097/ACM.0000000000000388, 24979285

[34] Bordin E. The generalizability of the psychoanalytic concept of the working alliance. Psychother Theory Res Pract. (1979) 16:252–60. doi: 10.1037/h0085885

[35] Burk-Rafel J Drake CB Sartori DJ. Characterizing residents’ clinical experiences-a step toward precision education. JAMA Netw Open. (2024) 7:e2450774. doi: 10.1001/jamanetworkopen.2024.50774, 39693075

[36] Wahlberg KJ Pay T Repp AB Wahlberg EA Kennedy AG. Effect of patient safety curriculum for internal medicine residents on a health care system. ATS Sch. (2022) 3:156–66. doi: 10.34197/ats-scholar.2021-0088IN, 35633999 PMC9131888

[37] Kennedy TJT Regehr G Baker GR Lingard LA. Progressive independence in clinical training: a tradition worth defending? Acad Med. (2005) 80:S106–11. doi: 10.1097/00001888-200510001-00028, 16199447

[38] Pomaki G Supeli A Verhoeven C. Role conflict and health behaviors: moderating effects on psychological distress and somatic complaints. Psychol Health. (2007) 22:317–35. doi: 10.1080/14768320600774561

[39] Varpio L Ray R Dong T Hutchinson J Durning SJ. Expanding the conversation on burnout through conceptions of role strain and role conflict. J Grad Med Educ. (2018) 10:620–3. doi: 10.4300/JGME-D-18-00117.1, 30619516 PMC6314365

[40] Dweck CS. Mindset: the new psychology of success. New York, NY: Random House (2006).

[41] Dweck CS Yeager DS. Mindsets: a view from two eras. Perspect Psychol Sci. (2019) 14:481–96. doi: 10.1177/1745691618804166, 30707853 PMC6594552

[42] Huffman BM Hafferty FW Bhagra A Leasure EL Santivasi WL Sawatsky AP. Resident impression management within feedback conversations: a qualitative study. Med Educ. (2021) 55:266–74. doi: 10.1111/medu.14360, 32815152

[43] Purdy E Borchert L El-Bitar A Isaacson W Jones C Bills L . Psychological safety and emergency department team performance: a mixed-methods study. Emerg Med Australas. (2023) 35:456–65. doi: 10.1111/1742-6723.14149, 36519387

[44] Ojute F Gonzales PA Berler M Puente N Johnston B Singh D . Investigating workplace support and the importance of psychological safety in general surgery residency training. J Surg Educ. (2024) 81:514–24. doi: 10.1016/j.jsurg.2023.12.010, 38388307

[45] Eldor L Hodor M Cappelli P. The limits of psychological safety: nonlinear relationships with performance. Organ Behav Hum Decis Process. (2023) 177:104255. doi: 10.1016/j.obhdp.2023.104255, 41253590

